# A Flexible Dual-Mode Photodetector for Human–Machine Collaborative IR Imaging

**DOI:** 10.1007/s40820-025-01758-5

**Published:** 2025-04-24

**Authors:** Huajing Fang, Xinxing Xie, Kai Jing, Shaojie Liu, Ainong Chen, Daixuan Wu, Liyan Zhang, He Tian

**Affiliations:** 1 Center for Advancing Materials Performance From the Nanoscale (CAMP‑Nano), State Key Laboratory for Mechanical Behavior of Materials, Xi’an Jiaotong University, Xi’an, 710049 People’s Republic of China; 2 School of Integrated Circuits and Beijing National Research Center for Information Science and Technology (BNRist), Tsinghua University, Beijing, 100084 People’s Republic of China

**Keywords:** MXene, Dual-mode IR imaging, Photothermoelectric, Thermochromic, Human–machine collaboration

## Abstract

Photothermoelectric (PTE) photodetectors with self-powered and uncooled advantages have attracted much interest due to the wide application prospects in the military and civilian fields. However, traditional PTE photodetectors lack of mechanical flexibility and cannot operate independently without the test instrument. Herein, we present a flexible PTE photodetector capable of dual-mode output, combining electrical and optical signal generation for enhanced functionality. Using solution processing, high-quality MXene thin films are assembled on asymmetric electrodes as the photosensitive layer. The geometrically asymmetric electrode design significantly enhances the responsivity, achieving 0.33 mA W^−1^ under infrared illumination, twice that of the symmetrical configuration. This improvement stems from optimized photothermal conversion and an expanded temperature gradient. The PTE device maintains stable performance after 300 bending cycles, demonstrating excellent flexibility. A new energy conversion pathway has been established by coupling the photothermal conversion of MXene with thermochromic composite materials, leading to a real-time visualization of invisible infrared radiation. Leveraging this functionality, we demonstrate the first human–machine collaborative infrared imaging system, wherein the dual-mode photodetector arrays synchronously generate human-readable pattern and machine-readable pattern. Our study not only provides a new solution for functional integration of flexible photodetectors, but also sets a new benchmark for human–machine collaborative optoelectronics.

**Supplementary Information:**

The online version contains supplementary material available at 10.1007/s40820-025-01758-5.

## Highlights

- An optical/electric dual-mode photodetector array for human–machine collaborative infrared imaging was demonstrated.
- The responsivity of the photothermoelectric photodetector was improved by the geometrically asymmetric electrodes.
- Coupling the photothermal conversion of MXene with thermochromic composites enabled a direct visualization of invisible infrared radiation.

**Supplementary Information:**

The online version contains supplementary material available at 10.1007/s40820-025-01758-5.

## Graphical abstract

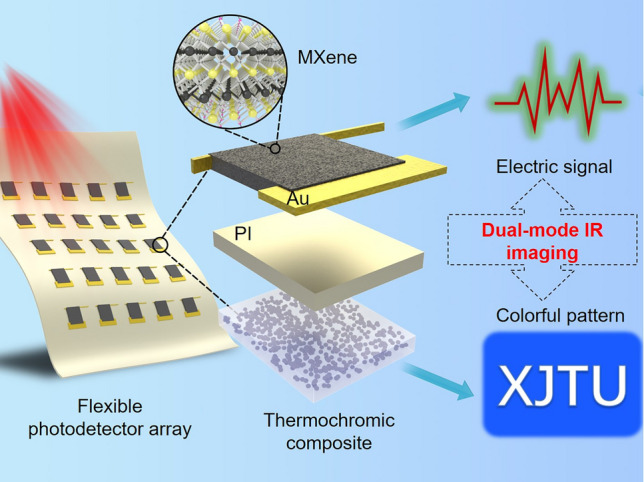

## Introduction

Infrared photodetectors are devices that convert incident infrared radiation signals into electrical signals, which have a wide range of applications such as military infrared guidance, security monitoring cameras, and medical thermal imaging diagnosis [1–4]. Infrared photodetectors utilize the physical effects presented by the interaction between infrared radiation and matter to detect the invisible light. According to the working mechanism of the devices, they can be divided into different classes including photoconductive detectors, photovoltaic detectors, quantum well detectors, bolometers, pyroelectric detectors and so on [5–8]. Among numerous infrared detecting mechanisms, the photothermoelectric (PTE) mode photodetectors are a kind of self-powered device that combine photothermal and thermoelectric effects. With the advantages of room temperature operability and low dark current, PTE photodetectors have become a research hotspot in recent years [9–12]. For example, Gong et al. designed a metasurface on a semimetallic Cd_3_As_2_ nanoplate to improve its thermoelectric photoresponse, yielding a responsivity of about 1 mA W^−1^ with the metasurface-enhanced light absorption [13]. Bao et al. developed a phonon-enhanced PTE photodetector with sensitivity up to 1.2 V W^−1^ and broadband spectral response from 325 nm to 10.67 μm based on reduced STO (SrTiO_3-δ_) single crystal [14]. Wang and co-workers proposed an infrared PTE photodetector with ultrahigh polarization sensitivity up to 2.5 × 10^4^, which was constructed on tellurium nanoribbon and perfect plasmonic absorber [15]. Despite these significant progress, infrared photodetectors based on the principle of PTE effect still have some issues to overcome. Inorganic materials that combine photothermal and thermoelectric effects are usually hard and brittle, lacking mechanical flexibility, which limits the application in flexible electronics and wearable devices. In this situation, there is an urgent need for flexible photodetectors that combine high detection performance and excellent mechanical performance [16–19]. Another drawback is that all photodetectors convert light into electrical signals, and without an electrical signal detection instrument, the device cannot work independently. In the wild or in some emergency situations, there is only a need to quickly and qualitatively determine the presence or absence of infrared radiation. However, the existing photothermoelectric photodetectors lack a convenient and visual recognition mode.

With the rapid development of technologies such as artificial intelligence and robotics, the requirements for photodetectors are also increasing. Various robots equipped with advanced sensors (including photodetectors) have been developed to work efficiently and reliably in environments that are not suitable for humans [20–22]. However, robots often perform poorly in situations that require perception, reasoning, and learning [23]. Intelligent sensing based on human–machine collaboration is expected to solve this bottleneck. It refers to the collaboration between humans and machines through intelligent sensors as a medium to achieve tasks such as perception, monitoring, and analysis of the environments [24–26]. Photodetectors are key sensors in the visual perception system of robots. It provides important visual information for robots by collecting light signals from the environment [27–29]. The human–machine collaborative visual signal processing is based on the rapid and accurate collection of data by photodetectors, and utilizes human cognitive abilities to further verify and supplement these data. Therefore, it can better adapt to the variable circumstances and complex tasks. Photodetectors that can simultaneously provide readable signals for machines and humans are the hardware foundation for implementing this concept. However, to the best of our knowledge, there are no relative reports on such infrared photodetectors up to now.

**Fig. 1:**
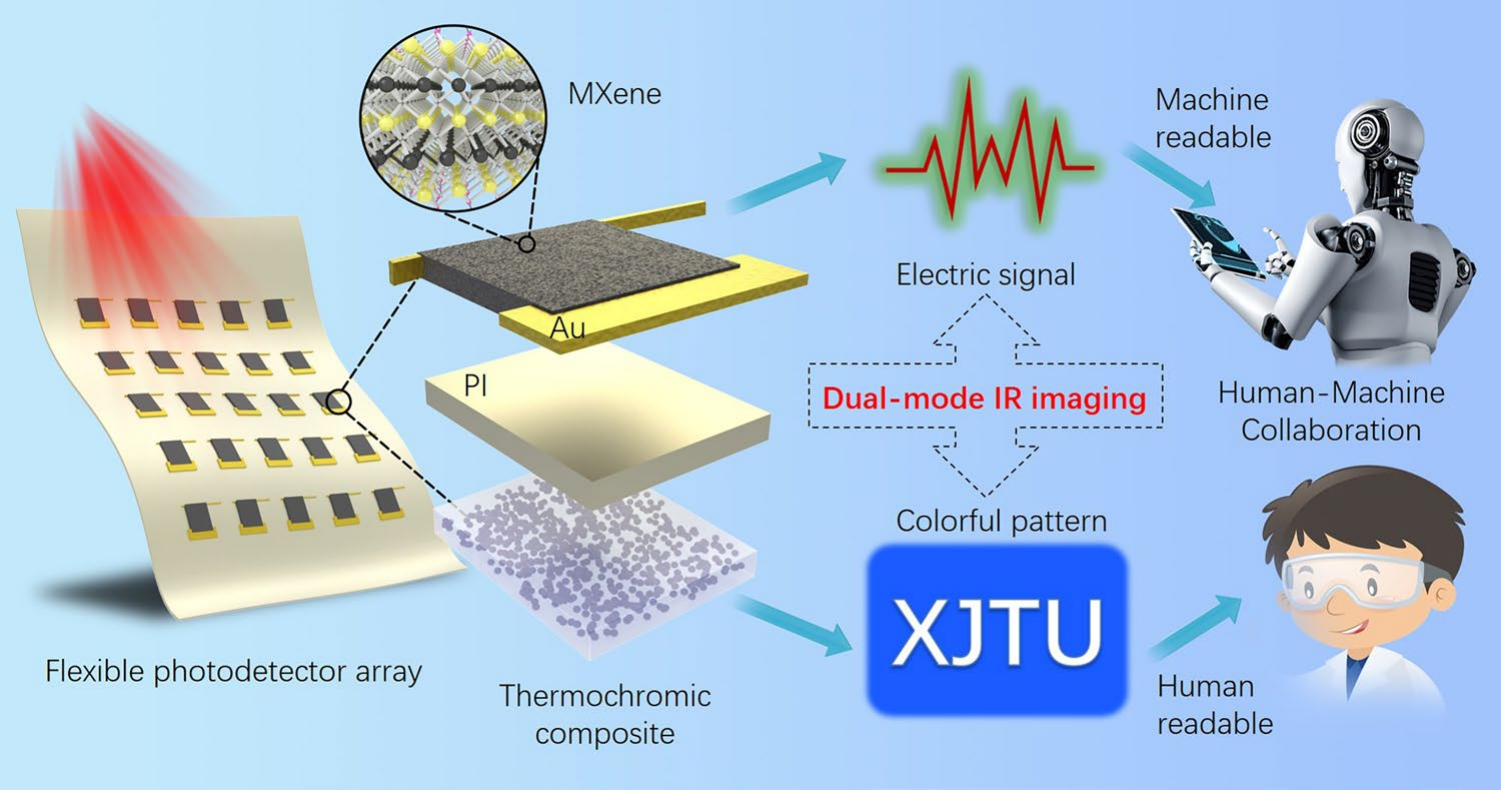
Schematic illustration of the flexible photodetector array for dual-mode IR sensing

In this work, we have developed a novel flexible dual-mode infrared photodetector as shown in Fig. 1. As a rising star two-dimensional (2D) material in optoelectronic devices, MXene represented by Ti_3_C_2_T_*x*_ (T stands for the surface terminations such as –O, –OH, and –F), has many advantages such as high conductivity, broad-spectrum absorption, and high photothermal conversion efficiency [30–34]. By utilizing the excellent PTE properties of 2D MXene materials, infrared radiation signals can be first converted into current signals quantitatively. The design of geometrically asymmetric metal electrodes greatly improves the PTE conversion efficiency of the device, thereby enhancing its infrared detection performance. At the same time, the good wettability of the gold electrode makes the interface contact resistance more stable, leading to the excellent mechanical flexibility of the photodetector. More importantly, by the coupling of photothermal and thermochromic effects, we provide a simple infrared visualization solution. This dual-mode photodetector can not only provide quantitative infrared detection through electrical mode for machine processing, but also provide intuitive detection through optical mode for human perception. Such a dual-mode IR imaging paradigm will promote the development of human–machine collaborative optoelectronic technology in the future.

## Materials and Method

### Materials

The Ti_3_C_2_T_x_ MXene nanosheets used in this study were obtained by selectively etching off the Al layer atoms from Ti_3_AlC_2_ MAX powder (Jilin 11 Technology Co., Ltd.). Specifically, 2 g of LiF (98%, Sinopharm Chemical Reagent Co., Ltd) was slowly added into 20 mL of concentrated hydrochloric acid (36%-38%, Sinopharm Chemical Reagent Co., Ltd) and stirred for 10 min in a PTFE beaker to completely dissolve it. Then 1 g of Ti_3_AlC_2_ MAX powder was slowly added to the above mixed solution and stirred in an oil bath at 40 °C for 24 h to completely etch away the Al layer. Subsequently, the reaction mixture was repeatedly washed by centrifugation with deionized water until pH ≥ 6. After washing, the precipitate obtained by centrifugation was added into 80 mL of deionized water, and ultrasonicated in an ice bath for 1 h to exfoliate the multilayer MXene. Finally, the ultrasonicated product was centrifuged at 3500 r min^−1^ for 1 h. MXene nanosheets dispersion was obtained in the collected supernatant.

Flexible circuit boards with polymide (PI) substrate were customized from Shenzhen Honghuifu Electronics Technology Co., Ltd. For symmetric PTE device, the widths of the two electrodes at both sides are 3 mm, and the channel between the electrodes is 3 mm. For asymmetric PTE device, the width of the two electrodes are 3 mm (wide side) and 0.1 mm (narrow side), respectively. And the channel between the electrodes is also 3 mm. The thermochromic composite material was fabricated by mixing the PDMS and thermochromic particles (Aobo Anti-counterfeiting Ink Technology Co., Ltd.) in a mass ratio of 10:1.

### Device Fabrication

Taking the asymmetric PTE device as an example, the flexible circuit board was first ultrasonically cleaned with deionized water. After cleaning, the flexible circuit board was further treated with air plasma to improve its surface hydrophilicity. To prepare MXene thin films of the specified size, Kapton tape was firstly adhered to the selected area of the flexible circuit board and formed a rectangle template with a length of 5 mm and a width of 4.5 mm. Then, 25 μL of the above MXene nanosheets dispersion was drop-casted inside the rectangle template and naturally dried to assemble the MXene thin film. The contact length between the MXene film and the wide electrode is 1.5 mm, while the narrow electrode is completely covered by the MXene film. Silver paste and silver wires were used for electrical connection during photoelectric measurement. The preparation process of photodetector arrays and symmetric PTE device is similar to that of the above asymmetric PTE device.

### Characterization

The morphology and micro-area composition of the MXene nanosheets were characterized by transmission electron microscopy (TEM, FEI Talos F200x) equipped with energy-dispersive X-ray spectroscopy (EDS). Fourier transform infrared spectroscopy (FTIR, Thermo Fisher Scientific Nicolet iS20) was used to analyze the composition of MXene nanosheets. The absorption spectrum of MXene aqueous suspension was measured by a spectrophotometer (Mapada V-1600PC). The thickness of the MXene film was measured by a step profiler (Bruker Dektak XT). The Thermal Imager (Fluke TiS75+) was used to measure temperature distribution of the samples and record the real-time temperature changes. The morphology of the MXene thin film and the thermochromic composite was observed by scanning electron microscope (SEM, ZEISS Sigma 360 and SEM, FEI Quanta FEG Series). All electrical measurements were conducted using a digital source meter (Keithley 2410). The 808 nm laser (MDL-III-808) was used as the point light source, and the power adjustable infrared LED is used as the surface light source. The optical power of the light source was calibrated by an optical power meter (VLP-2000-2W). Thermal conductivity measurements of samples were performed on the Hot Disk method (Hot Disk TPS2500S).

## Results and Discussion

### Characterization and Photothermal Performance of MXene

**Fig. 2:**
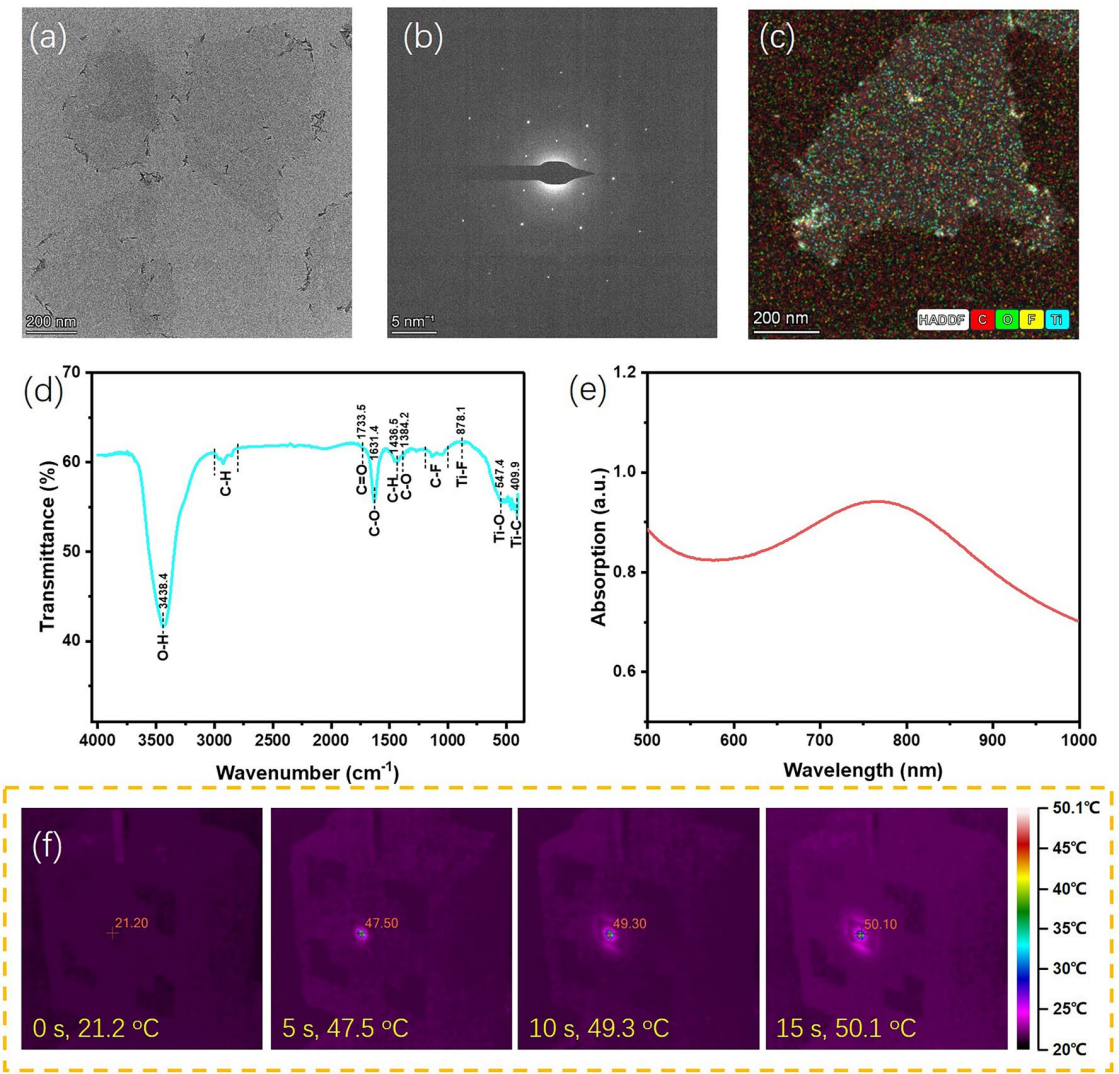
**a** TEM image of the Ti_3_C_2_T_*x*_ MXene nanosheets. **b** SAED image of the Ti_3_C_2_T_*x*_ MXene nanosheet. **c** HADDF and EDS mapping of a single MXene nanosheet. **d** FTIR of the prepared Ti_3_C_2_T_*x*_ MXene nanosheets. **e** Absorption of the Ti_3_C_2_T_*x*_ MXene aqueous suspension. **f** Fast photothermal conversion of the MXene thin film

Figure 2a shows the transmission electron microscope (TEM) image of the Ti_3_C_2_T_*x*_ nanosheets prepared through lithium ions intercalation and ultrasonic bath. Ultrathin nanosheets with lateral size around several hundred nanometers confirm the two-dimensional morphology of MXene. The small particles at the edges of the nanosheets may be due to slight oxidation of MXene during the preparation process. Figure 2b shows the selected area electron diffraction (SAED) of a Ti_3_C_2_T_*x*_ MXene nanosheet. A typical hexagonal symmetric diffraction pattern can be observed in the SAED image, indicating the good crystallinity of the prepared MXene nanosheets [35]. Elemental analysis of the selected individual nanosheet can provide compositional information. Figure 2c shows the overlap high-angle annular dark-field (HAADF) scanning TEM and energy-dispersive X-ray spectroscopy (EDS) elemental mapping image. The C, O, F, and Ti elements are evenly distributed throughout the nanosheet. Among them, Ti and C come from the main components of Ti_3_C_2_T_*x*_ MXene, while O and F come from the surface functional groups such as –F and –OH. The high-quality MXene nanosheets have laid the foundation for us to prepare flexible devices. The FTIR spectra of the prepared MXene nanosheets is shown in Fig. 2d, in which many fingerprint peaks can be clearly observed. These fingerprint peaks are referred to the vibration mode such as O–H stretching, C–H stretching and so on [36]. The results of FTIR are consistent with EDS elemental mapping, further confirming the presence of surface functional groups. Figure 2e presents the absorption spectra of MXene aqueous suspension at the wavelength range of 500 to 1000 nm. Deducting the absorption background of pure water, the MXene nanosheets exhibit a high basic absorption with an absorption peak around 800 nm. Based on the solution to Maxwell equation, a conductive material with low resistance usually leads to high extinction coefficient as well as the good electro-magnetic wave absorption [37]. MXene, as a low resistance two-dimensional material, can even replace metals as electrode materials in energy and optoelectronic devices [38, 39]. Hence, the associated high light absorption makes MXene an ideal photothermal material for biomedical application and solar energy utilization [40, 41]. In order to verify the superior photothermal conversion ability of the prepared MXene nanosheets, we monitored the temperature changes of MXene thin films in real-time during infrared irradiation. The thickness of the MXene film was measured using a step profiler, yielding a thickness of 15 μm (see Fig. S1). As shown in Fig. 2f, the initial temperature is 21.2 °C, close to room temperature. After 5 s of infrared irradiation, the temperature rapidly reached 47.5 °C, with the average heating rate above 5 °C s^−1^. During the 10 s continuous exposure to light, the temperature tends to saturate. Such a good photothermal conversion capability is exactly what PTE devices require. We also measured the temperature coefficient of resistance (TCR) of the MXene film, to elucidate the effect of heating on their electrical properties. As shown in the I-V curves of Fig. S2, the resistances obtained at 30 and 70 °C are 9.321 and 9.125 Ω, respectively. Hence, the calculated TCR of MXene film is 5.3 × 10^−2^% °C^−1^, comparable to the values in previous work [42].

### Enhanced Photothermoelectric Performance by Asymmetric Electrodes

**Fig. 3:**
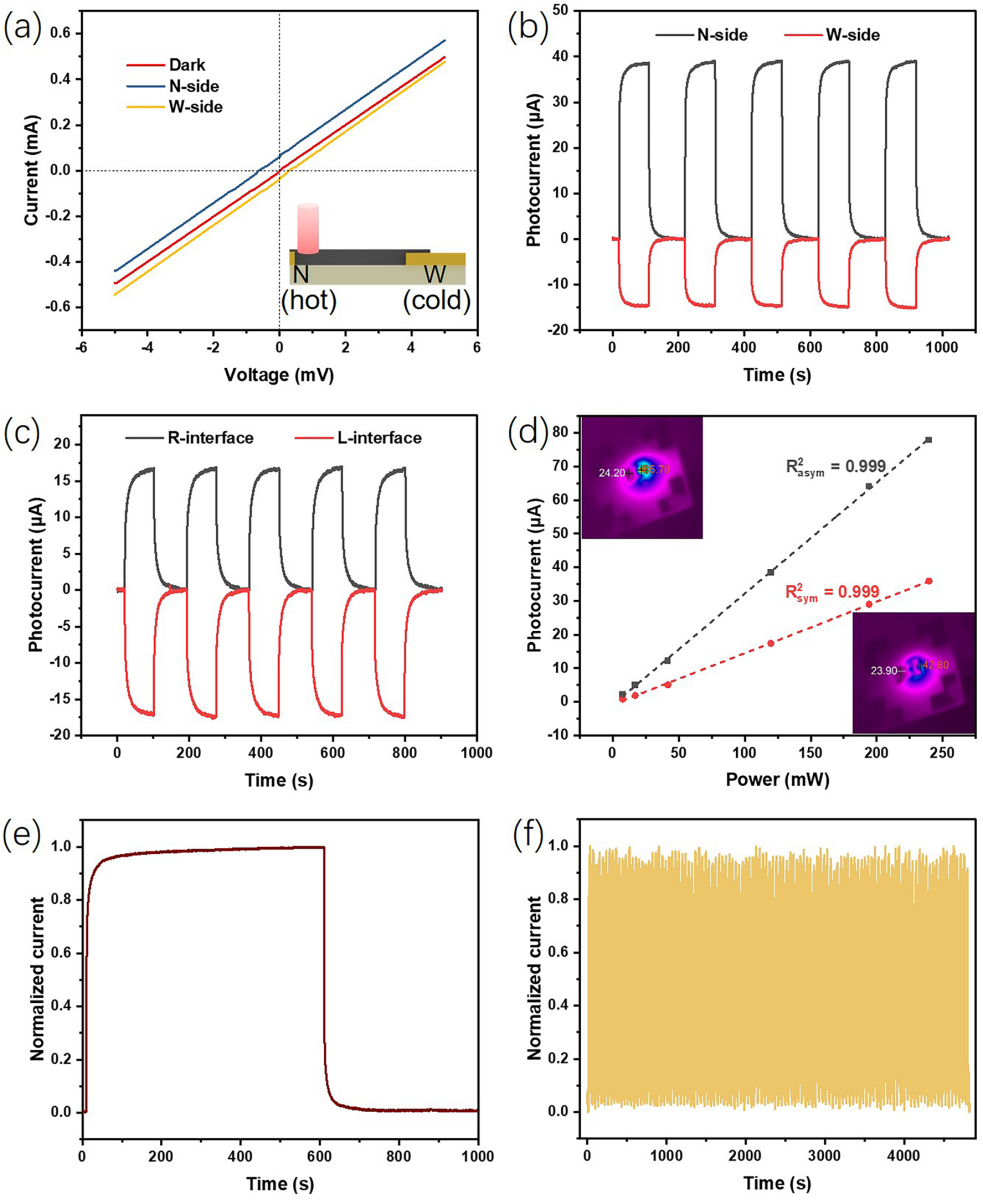
**a** I–V curves of the asymmetric PTE photodetector in the dark and under illumination of 808 nm IR light, inset shows the working mechanism of asymmetric PTE photodetector. **b** Time responses of the asymmetric PTE photodetector under the periodic illumination of the 808 nm laser, the light spots are, respectively, irradiated on wide electrode side and the narrow electrode side. **c** Time responses of the symmetric PTE photodetector under the periodic illumination of the 808 nm laser, the light spots are, respectively, irradiated on left and right side. **d** Photocurrent as the function of illumination intensity (0.236–7.63 W cm^−2^) with 0 V bias for both PTE photodetectors, insets are the infrared thermal images of the symmetric and asymmetric devices. **e** Photocurrent curve of the asymmetric PTE device kept in ambient air under more than 10 min illumination. **f** Long-term operational stability of the asymmetric PTE device

The inset of Fig. 3a depicts the construction and detection mechanism of the asymmetric PTE photodetector. The width of the gold electrodes on both sides is different. In the following text, N side and W side are used to refer to narrow and wide electrodes, respectively. We verified the thermoelectric performance of the device through a simple experiment. By heating only one side of the narrow electrode with a heated iron rod, the current change of the device is shown in Fig. S3. When the heated iron rod approached the narrow electrode, the device’s current rapidly increased, while the rod was moved away, the current quickly decreased. This current change clearly demonstrates the Seebeck effect of the asymmetric PTE device. When the IR laser spot is placed on the N side, the local temperature increases due to the photothermal conversion effect of MXene. Hence, carriers migrate from the N side (hot end) to the W side (coldend) following the Seebeck effect. The three I–V curves in Fig. 3a are obtained in the dark and illuminated at N/W side, respectively. The linear I–V behavior of all three curves indicates the Ohmic contact between the MXene thin and Au electrodes. The intersection point between the two curves under illumination and the horizontal axis is the open-circuit voltage (*V*_oc_), which also indicates that the device can operate in self-powered mode. Obviously, the open-circuit voltage obtained by the laser spot on the N side is greater than that on the W side. Subsequently, the output current of the device was tested under zero bias voltage and periodic illumination of the 808 nm laser. Although the light intensity is the same, the resulting current values vary greatly when the laser spots are, respectively, irradiated at N side and W side. From a single on–off switching cycle as shown in Fig. S4, we can obtain the response speed of the asymmetric PTE photodetector. The rise time *t*_r_ and fall time *t*_f_ of the asymmetric device are 8.53 and 7.39 s, respectively, for N side illumination. Although the response speed of PTE photodetector may be relatively slower than that of photoelectric conversion type photodetector, it is already meet the actual requirements for the application of human–machine collaborative infrared imaging in this work. On the contrary, the current generated by the PTE device with symmetrical electrodes is almost equal in magnitude as shown in Fig. 3c. Comparing the results in Fig. 3b, c, it can be found that the geometric asymmetry of the Au electrodes significantly increases the output current of the PTE device. To investigate the relationship between asymmetry and current response, we conducted additional testing on an intermediate sample with one 3-mm-wide electrode and another 1-mm-wide electrode. Compared to the symmetric electrodes (both 3 mm) and the asymmetric electrodes (3 and 0.1 mm), its degree of asymmetry falls between these two configurations. Photocurrent of the three devices were tested under identical illumination conditions. As shown in Fig. S5, the symmetric (both 3 mm) electrode device exhibits the lowest photoresponse, while the asymmetric electrode (3 and 0.1 mm) device demonstrates the highest photoresponse. The photocurrent of the intermediate sample (3 and 1 mm) is between the previous two devices. Therefore, we can conclude that the higher degree of electrode asymmetry, the greater the photoresponse of the device.

We further tested the photocurrent under different light power and fitted the results in Fig. 3d. The relationship between the photocurrent and the incident light power of both PTE devices shows an almost perfect linear correlation. This linear feature enables PTE devices as photodetectors to conveniently monitor light intensity. Under different light intensities, the photocurrent of asymmetric PTE device is always greater than that of symmetric device, and the advantage becomes more prominent under strong light. This phenomenon can be attributed to the expanded temperature gradient inside the asymmetric PTE device. We used an infrared camera to capture the temperature distribution of two devices when they reached thermal equilibrium. In the insets of Fig. 3d, we provide infrared photographs of symmetric and asymmetric devices under 808 nm laser irradiation, which visualize the temperature distribution on each device. The upper-left inset reveals that irradiating the narrow electrode of the asymmetric device generates a maximum steady-state temperature of 85.7 °C at the irradiation point, while maintaining the wide electrode as the low-temperature end at 24.2 °C (Δ*T* = 61.5 °C). In contrast, the lower-right inset shows the symmetric device with laser irradiation at its right end, achieving only 42.8 °C at the irradiated spot (half the asymmetric device’s temperature) and 23.9 °C at the left low-temperature end (Δ*T* = 18.9 °C). These infrared thermal images visually validate that the asymmetric electrode design significantly enhances the temperature gradient. As shown in Fig. S6, when laser irradiation is applied to the junction between MXene and the Au electrode at one end of the device, the temperature at the interface increases due to MXene’s photothermal conversion capability. Subsequently, charge carriers migrate from the hot end to the coldend under the Seebeck effect, generating current in the circuit. However, the Au electrodes also act as heat sinks. Under the same laser intensity, the narrow electrode in the asymmetric device has a smaller heat dissipation area compared to the symmetric device, resulting in a higher temperature at the narrow electrode. Since the cold-end temperatures of both devices are approximately equal, the temperature gradient in the asymmetric device is greater than that in the symmetric device. According to the Seebeck effect, it can be inferred that larger temperature gradient naturally results in greater electrical output. Responsivity (*R*) is a key indicator for evaluating the performance of photodetectors, which can be calculated as follow:

1 $$R = I_{{{\text{ph}}}} /P_{{{\text{in}}}}$$

where *I*_ph_ is the photocurrent and *P*_in_ is the incident light power. By calculating the slope in Fig. 3d, the responsivity of the symmetric PTE photodetector is 0.15 mA W^−1^. Through a simple geometric asymmetric electrode design scheme, we have doubled the responsivity of the PTE photodetector to 0.33 mA W^−1^. The simple solution processing technology combined with clever design scheme significantly improves the responsivity of the PTE device, which is comparable to the values in previous works [43, 44].

**Fig. 4:**
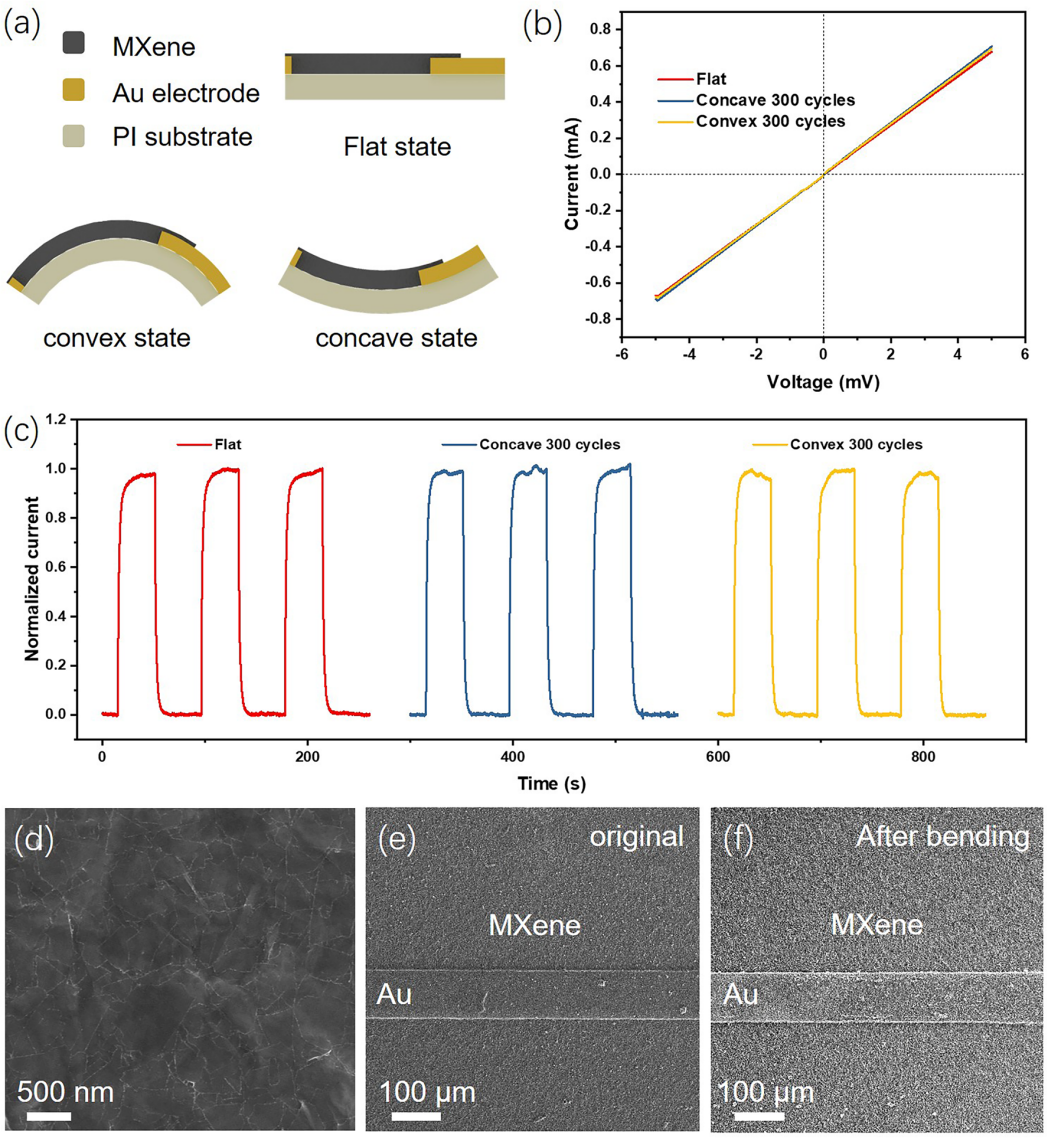
**a** Schematic illustration of bending test of the flexible PTE photodetector based on MXene film and asymmetric Au electrodes. **b** I–V curves of the PTE photodetector under different bending condition. **c** Output photocurrent of the PTE device at original state and after long-term concave/convex bending test. **d** SEM image of the prepared MXene thin film on the PI substrate. **e** and **f** SEM images of the area near the narrow Au electrode before and after bending test

The stability of the photodetector is an important factor affecting the performance of the device in practical environments. To evaluate the stability of the MXene-based PTE device, we tracked and tested the current signal under different illumination conditions. As the normalized current in Fig. 3e displays, the output signal remains almost constant during the continuous illumination of more than 10 min. It is a direct evidence to demonstrate the light-soaking stability of the asymmetric PTE photodetector. In addition, we have also checked the long-term operational stability of the asymmetric PTE photodetector, as shown in Fig. 3f. Under intermittent illumination conditions exceeding one hundred cycles, there is almost no fatigue attenuation of current signal, even if the sample is exposed to air. The advantage of stability makes the asymmetric PTE photodetector more practical.

### Excellent Flexibility of the PTE Photodetector

When the thickness of MXene nanosheet is reduced to the atomic scale, its mechanical properties are significantly different from those of bulk MAX phase materials. The unique 2D structure of MXene nanosheet makes it an excellent choice for constructing flexible electronics [45–47]. The flexibility of our asymmetric PTE device has been investigated under different bending conditions as shown in Fig. 4a. The original device is in a flat state, then we applied external force to make it in convex state or concave state. The bending strain of the entire photodetector can be calculated using the formula *ε* = *D*/*ρ*, where *D* and *ρ* denote the half-thickness of the device and the bending radius, respectively. Figure 4b shows the I–V curves of the asymmetric PTE device in the flat state and after undergoing 300 convex or concave bending cycles with a strain of 0.31%. The almost overlapping three straight lines indicate that the electrical performance of the device has not changed after bending test. From the slope in Fig. 4b, it can be calculated that the overall resistance of the device is only 7.3 Ω, including the intrinsic resistance of MXene thin film and the interfacial contact resistance. It is worth mentioning that in PTE devices, the internal resistance is fatal to the output current value. Generally, in order to obtain the ideal output current, it is necessary to minimize the internal resistance of the PTE material as much as possible. From this perspective, MXene thin films with excellent conductivity are indeed suitable for preparing PTE devices. Figure 4c shows the photocurrent of the PTE device at original state and after long-term concave/convex bending test. As expected, the analysis of the three photo response curves indicates negligible variance across the different bending conditions tested. To explore the source of excellent flexibility in the PTE device, we used scanning electron microscopy to observe its microstructure. As shown in Fig. 4d, the MXene nanosheets are stacked on top of each other, assembling into a uniform and flat thin film during the natural drying process. This uniform morphology ensures excellent conductivity and ideal flexibility inside the MXene thin film. The interface between the Au electrode and the MXene thin film is a dangerous zone where devices are prone to failure. Figure 4e is the SEM image observed from the area near the narrow Au electrode. The narrow Au electrode is 100 μm wide and uniformly covered by MXene thin film. After bending test, such morphology has not undergone significant changes. As shown in Fig. 4f, no microcracks, wrinkles and other defects are observed. These findings highlight the potential of our flexible asymmetric PTE photodetectors as wearable electronics. In order to more intuitively demonstrate the comprehensive performance of the device, the comparison of various performance indicators of the device with previous works are listed in Table S1 and Fig. S7. The asymmetric PTE device exhibits superior comprehensive performance compared to other reported devices, manifested in its self-powered operation, good responsivity, flexibility, and excellent operational stability.

### Dual-Mode IR Imaging

**Fig. 5:**
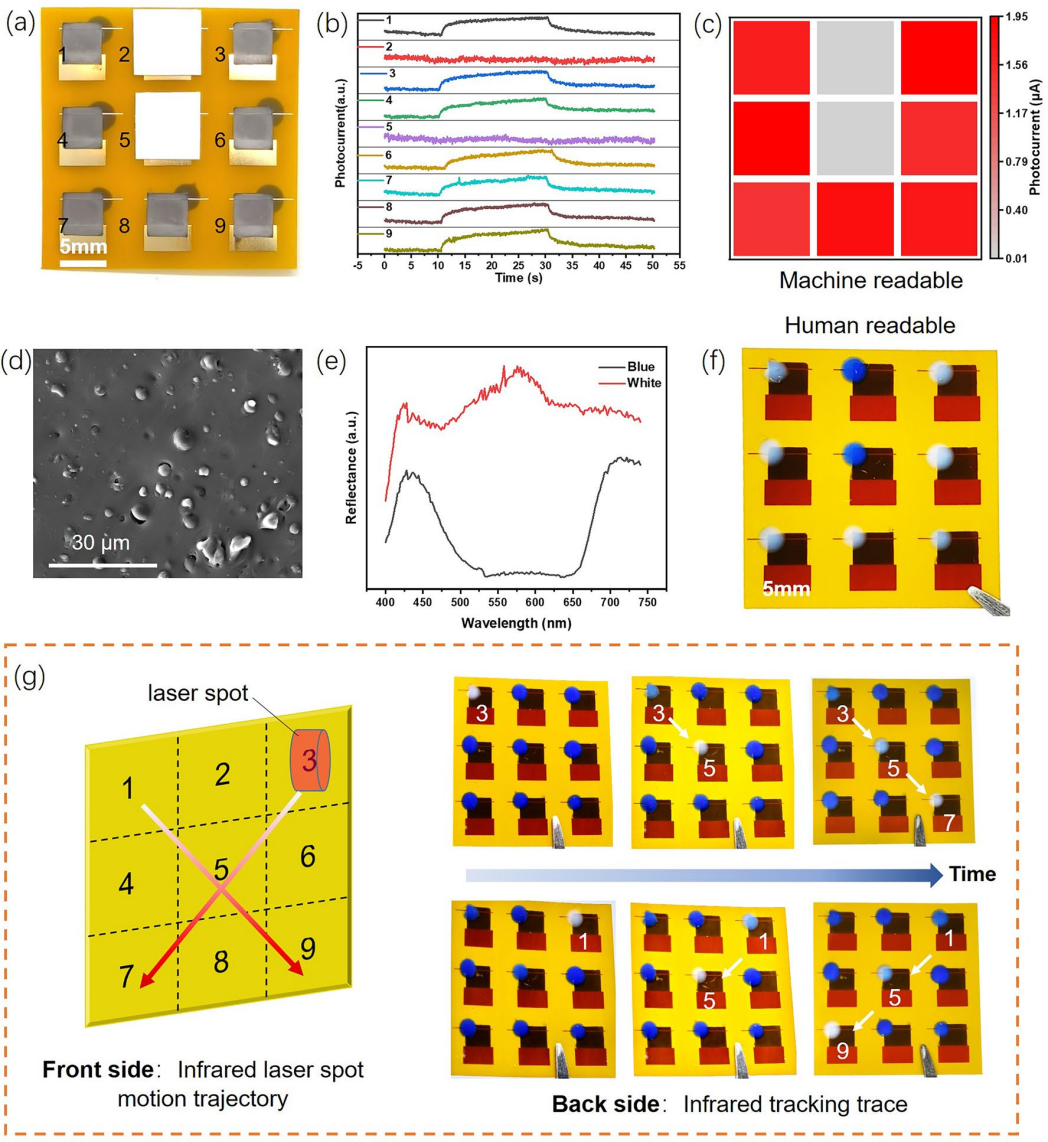
**a** Front side photograph of the 3 × 3 photodetector array with two obscured array elements. **b** Current output signals monitored from the nine array elements. **c** Infrared imaging obtained from current signal mapping processing. **d** Typical SEM image of the thermochromic composite. **e** Reflection spectra of the two states before and after thermochromic conversion. **f** Back side photograph of the 3 × 3 photodetector array with human-readable pattern. **g** Schematic diagram of infrared tracing application and photographs of infrared tracking trace

Next, we put the flexible PTE device into practical use of human–machine collaborative infrared application. The dual-mode IR imaging performance of the photodetector array has been verified by identifying the given patterns. Figure 5a shows the photograph of a 3 × 3 photodetector array taken from the front. The white square photomasks were placed above the photodetector array to block two of the array elements. So that a predetermined U-shaped pattern is formed under the illumination of a surface light source. For machine read mode, electric signals can be obtained by monitoring the output current of each array element. The current signals from the nine array elements were recorded and compared in Fig. 5b. Among them, no significant current fluctuations were detected in the 2# and 5# elements. While the current signals of the remaining seven samples were similar, showing good array homogeneity. After establishing a mapping relationship between the photocurrent signal and the two-dimensional coordinates of the array elements, the machine-readable “U” pattern was obtained as shown in Fig. 5c.

For the human read mode, invisible infrared signals must be converted into color changes that can be perceived by human eyes. To achieve this goal, we set the thermochromic composite material on the back of the flexible array. The composite materials consist of thermochromic particles with an average size of several micrometers and PDMS matrix, as shown in Fig. 5d. To investigate the effect of thermochromic particles on the device's thermal conductivity, thermal conductivity measurements were performed on both the thermochromic composite and pure PDMS matrix at 25 °C using the Hot Disk method. The results revealed that the composite exhibited a thermal conductivity of 0.2245 W m^−1^ K^−1^, compared to 0.1835 W m^−1^ K^−1^ for the PDMS matrix, indicating that microscale thermochromic particles enhances the thermal conductivity of PDMS. This composite material appears blue at low temperatures and turns white at high temperatures. Figure 5e depicts the reflectance spectra of composite materials in two states. In the blue state, there are two reflectance peaks around 430 and 720 nm. While in the white state, the composite material has high reflectance throughout the visible light spectrum. This distinct reflection state will create a vivid color contrast. As a result, the colorful “U” pattern can be directly seen with the naked eye from the back of the photodetector array. By comparing the imaging data in Fig. 5c, f, the recognition conclusions of humans and machines are consistent, further improving the accuracy of the imaging system. The similar dual-mode imaging function also performs smoothly in the recognition process of letter “X” pattern, as shown in Fig. S8. It can be expected that when facing more complex imaging tasks, combining the advantages of human and machine can fully unleash the potential of this human–machine collaborative imaging system.

In the photodetector array, the thermochromic composite materials on the back of each element can change their color along the irradiation path of the infrared laser, and gradually revert to their original color over time. Therefore, infrared tracking can visualize the position and timeliness of infrared laser irradiation through the coloring location and color intensity, enabling precise localization of the infrared trajectory. As illustrated in Fig. 5g, when the infrared laser moved diagonally across the array, the thermochromic materials of the diagonal elements sequentially transition from blue to white, then gradually revert to their original blue color over time. By analyzing the temporal sequence of color changes, we can determine the laser’s movement direction: from top-right to bottom-left (element 3# → 5# → 7#) and top-left to bottom-right (element 1# → 5# → 9#). Hence, the flexible PTE device can is not only capable of static IR imaging, but also capable of handling dynamic tracking tasks. Moreover, this dual-mode imaging system has higher energy utilization efficiency compared with traditional PTE devices. As we know that, the principle of PTE photodetector is based on the photothermal conversion and Seebeck effect. The energy of incident photons is first converted into thermal energy, and then converted into electrical energy through thermoelectric conversion. Besides this inherent energy utilization pathway, thermochromic materials can convert thermal energy into chemical energy to achieve color change. Therefore, a new energy conversion process has been introduced to the dual-mode devices.

## Conclusions

In summary, we have demonstrated the first human–machine collaborative infrared imaging system based on the dual-mode photodetector. The combination of high-quality MXene thin films and asymmetric Au electrodes endows the photodetector with excellent PTE performance and stability. The responsivity of the asymmetric PTE photodetector reached 0.33 mA W^−1^, which is twice as high as that of symmetric device. Concave or convex bending for 300 cycles does not affect the photo response of such PTE device. This excellent mechanical flexibility makes the device expected to play an important role in wearable electronics. Moreover, by integrating with thermochromic composite materials, the heat generated through photothermal conversion of MXene can trigger color changes for visual recognition. Hence, the dual-mode photodetector synchronously provides machine-readable electrical signals and human-readable optical signals. Consequently, this study shows a new concept of functional integrated infrared photodetector, which is expected to open up new avenues for human–machine collaborative vision systems.

## Supplementary Information

Below is the link to the electronic supplementary material.

Supplementary file1 (DOC 2074 KB)
