# Supplementary material for: A Flexible Dual-Mode Photodetector for Human–Machine Collaborative IR Imaging
Source: Nanomicro Lett. 2025 Apr 24;17:229. doi: 10.1007/s40820-025-01758-5 (PMC12021759; doi:10.1007/s40820-025-01758-5)
Supplement: Supplementary file 1 — Supplementary file1 (DOC 2074 KB) [file 40820_2025_1758_MOESM1_ESM.doc]

Supporting Information for

**A Flexible Dual-Mode Photodetector for Human-Machine Collaborative IR Imaging**

Huajing Fang^1^,*, Xinxing Xie^1^, Kai Jing^1^, Shaojie Liu^1^, Ainong Chen^1^, Daixuan Wu^2,*^, Liyan Zhang^1^ and He Tian^2^,*

^1^Center for Advancing Materials Performance From the Nanoscale (CAMP‑Nano), State Key Laboratory for Mechanical Behavior of Materials, Xi’an Jiaotong University, Xi’an 710049, P. R. China

^2^School of Integrated Circuits and Beijing National Research Center for Information Science and Technology (BNRist), Tsinghua University, Beijing 100084, P. R. China

*Corresponding authors. E-mail: [fanghj@xjtu.edu.cn](mailto:fanghj@xjtu.edu.cn) (Huajing Fang); [wxyjqm@yeah.net](mailto:wxyjqm@yeah.net) (Daixuan Wu); [tianhe88@tsinghua.edu.cn](mailto:tianhe88@tsinghua.edu.cn) (He Tian)

**Supplementary Figures and Tables**

**Fig. S1** The thickness of the MXene film


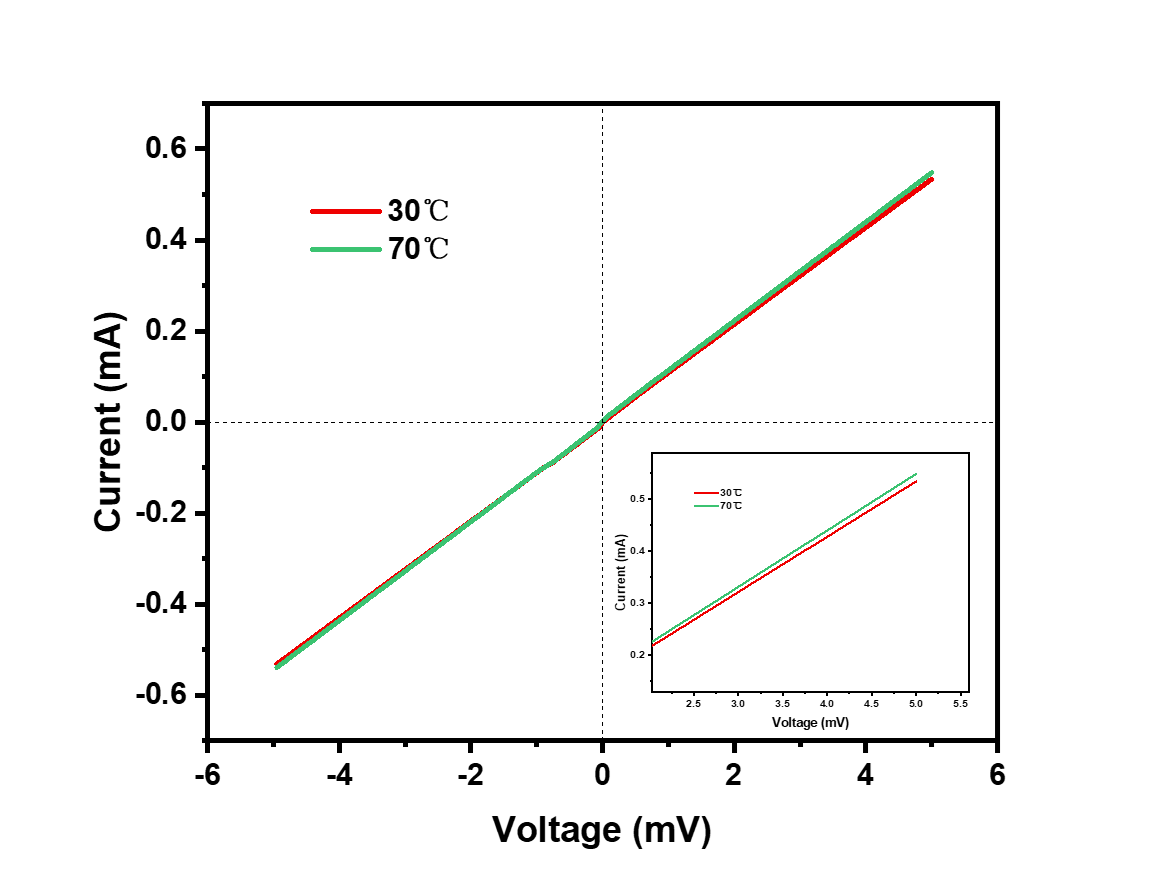


**Fig. S2** I-V curves of MXene film at 30 ℃ and 70 ℃

**Fig. S3** Current change of the device during heating and cooling narrow electrode

**Fig. S4** The rise time and fall time of asymmetric device for N-side illumination

**Fig. S5** Photocurrent of the three devices with different electrode configurations

*
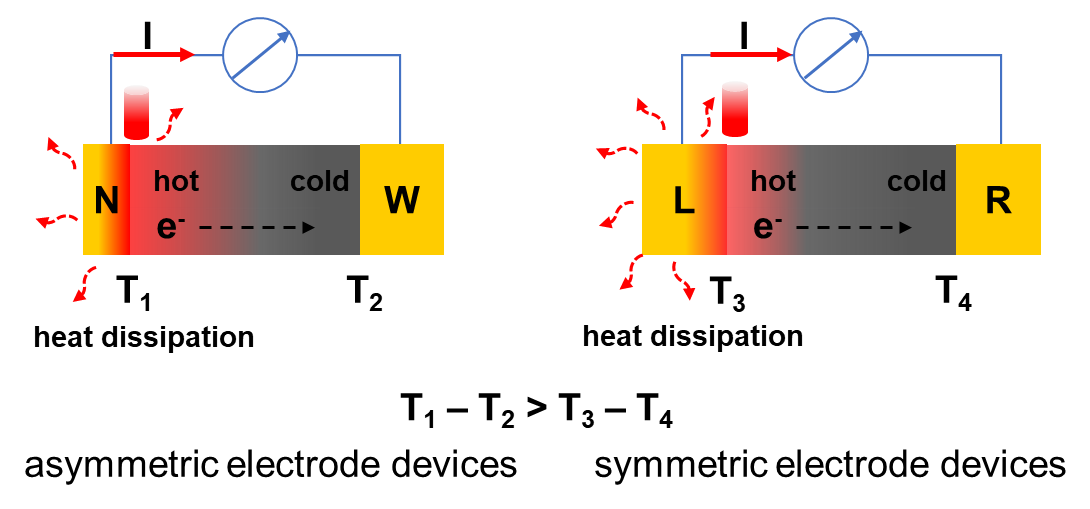
*

**Fig. S6** The working mechanism of asymmetric and symmetric PTE photodetector

***Table S1*** *comparison with previously reported work with figure of merits of the sensors*

| **Materials** | **Bias**  **Voltage (V)** | **Responsivity**  **(mA/W)** | **Response**  **time** | **Flexibility** | **Operational**  **Stability** (cycles) | **Refs.** |
| --- | --- | --- | --- | --- | --- | --- |
| MXene | 0 | 0.33 (808 nm) | 8.53 s | Yes | 300 | This work |
| WSe_2_/Ag | 1 | 0.43 (780 nm) | 4.1 s | Yes | 20 | [S1] |
| Bi_2_Te_3_ | 0 | 0.388 (8 μm) | 10 ms | No | 50 | [S2] |
| WSe_2_/CuO | 2 | 0.28 (700 nm) | 5.4 s | Yes | 1000 | [S3] |
| SnS/Mg | 10 | 52 (470 nm) | 3.9 s | Yes | 50 | [S4] |
| Graphene/Al | 0 | 0.25 (825 nm) | / | No | / | [S5] |
| SWCNTs/MoS_2_ | 0 | 0.088  (532 nm) | 102 ms | No | / | [S6] |

**Fig. S7** Radar chart to compare the comprehensive metrics of photothermoelectric detectors based on MXene, ZnO [S7] and Bi_2_Te_3_ [S2]


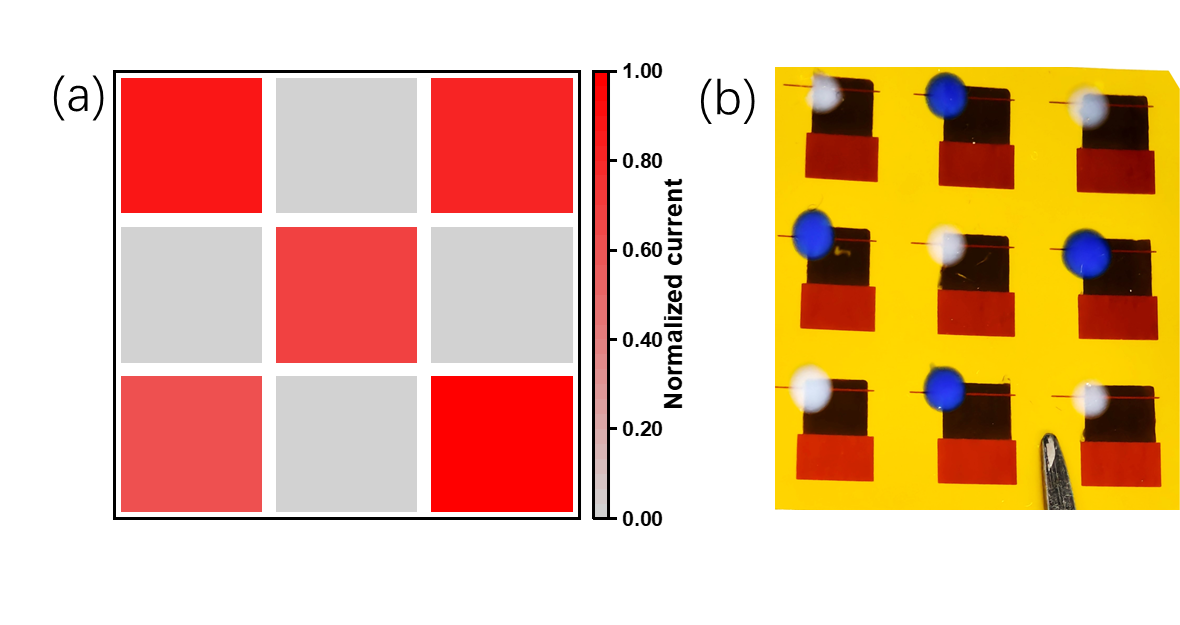


**Fig. S8** Image of “X”pattern from (**a**) machine read mode (**b**) human read mode

**Supplementary References**

1. R. P. Patel, P. M. Pataniya, M. Patel, K. Joshi, K. H. Modi et al., Hand-Print method for preparation of large area and binder free electrodes for photodetection and electrocatalytic hydrogen evolution. Sol. Energy **246**, 343-354 (2022). <https://doi.org/10.1016/j.solener.2022.09.016>
2. Y. Zhang, J. Jiang, Z. Zhang, H. Yu, Y. Lian et al., Long-wave infrared photothermoelectric detectors with resonant nanophotonics. J. Mater. Chem. C **12**, 16714–16721 (2024). <https://doi.org/10.1039/D4TC02504K>
3. R. P. Patel, P. M. Pataniya, M. Patel, V. Adepu, P. Sahatiya et al., Highly flexible and foldable broad band WSe_2_/CuO heterostructure photodetector. Sensor Actuat A-phys **356**, 114339 (2023). <https://doi.org/10.1016/j.sna.2023.114339>
4. P. V. Shah, P. Pataniya, N. N. Som, V. Sathe, S. Ck, Flexible and hand-printed photodetector based on Mg–SnS nanoflakes. ACS Appl. Nano Mater. **7**, 5967-5981 (2024). <https://doi.org/10.1021/acsanm.3c05876>
5. D. Wang, A. E. L. Allcca, T. F. Chung, A. V. Kildishev, Y. P. Chen et al., Enhancing the graphene photocurrent using surface plasmons and a p-n junction. Light Sci. Appl. **9**, 126 (2020). <https://doi.org/10.1038/s41377-020-00344-1>
6. P.-Y. Huang, H.-J. Chen, J.-K. Qin, L. Zhen, C.-Y. Xu. A polarization-sensitive photothermoelectric photodetector based on mixed-dimensional SWCNT–MoS_2_ heterostructures. Nanoscale Adv. **4**, 5290-5296 (2022). <https://doi.org/10.1039/D2NA00609J>
7. K. Zhang, B. Ouyang, Y. Wang, Y. Xia, Y. Yang, Coupling Enhancement of Photo-Thermoelectric Conversion in a Lateral ZnO Nanowire Array. ACS Appl. Energy Mater. **2**, 7647−7654 (2019). <https://doi.org/10.1021/acsaem.9b01633>
